# Supplementary material for: Architecture of the Sema3A/PlexinA4/Neuropilin tripartite complex
Source: Nat Commun. 2021 May 26;12:3172. doi: 10.1038/s41467-021-23541-x (PMC8155012; doi:10.1038/s41467-021-23541-x)
Supplement: Supplementary file 1 — Supplementary information [file 41467_2021_23541_MOESM1_ESM.pdf]

**Supplemental Information**

**Architecture of the Sema3A/PlexinA4/Neuropilin tripartite complex**

**Lu et al**

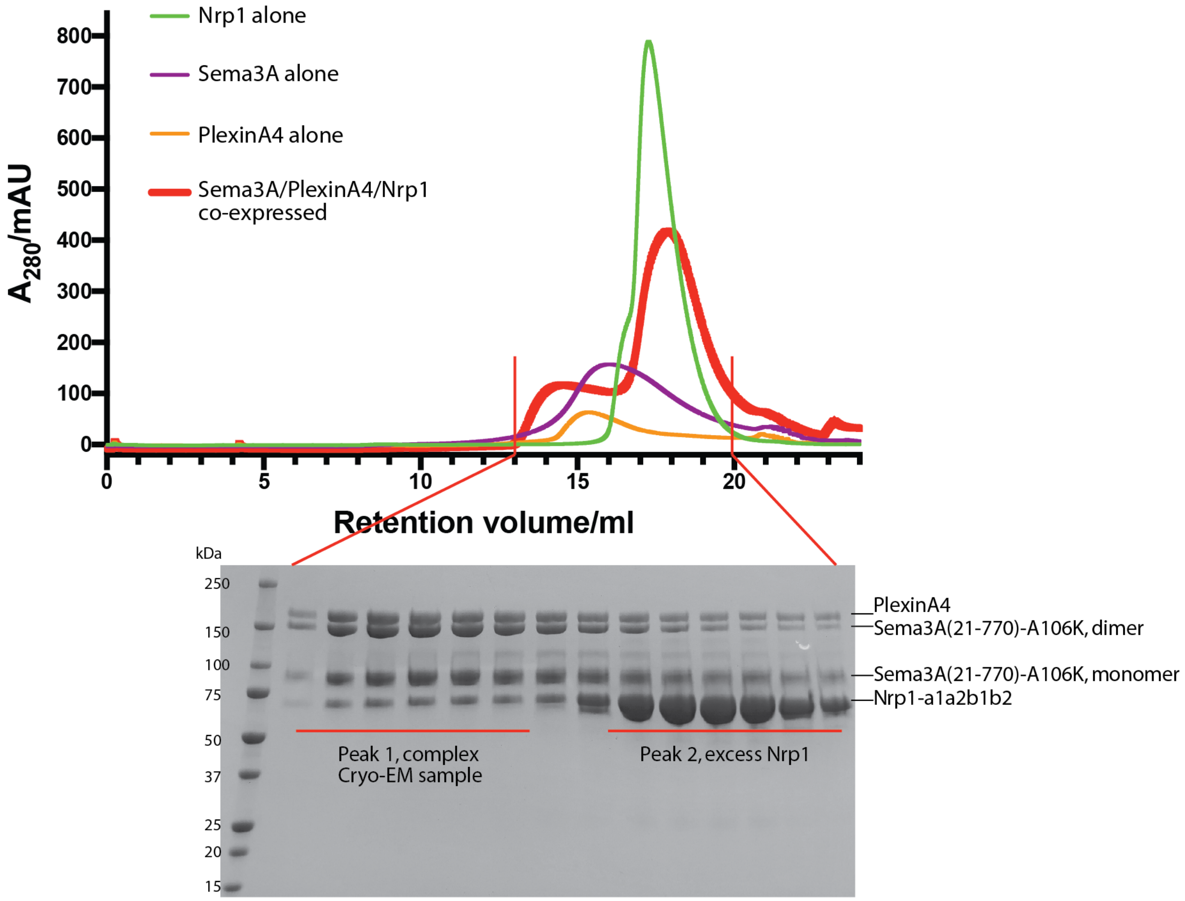


**Supplementary figure 1. Sample preparation of the Sema3A/PlexinA4/Nrp1 complex for Cryo-EM analyses**

The upper panel shows the elution profile on a Superose 6 10/30 column of the complex of Sema3A(21-770)-A106K, the PlexinA4 extracellular region and Nrp1-a1a2b1b2 from co-expression. The first peak is the complex, while the second is excess Nrp1, which expressed much better than Sema3A and PlexinA4. Gel filtration traces of the individual protein are shown for comparison. The lower panel shows SDS-PAGE analyses of the fractions from the gel filtration run of the complex sample. Note a portion of Sem3A(21-770)-A106K ran as a dimer on the gel, likely due to incomplete reduction of the disulfide bond formed by Cys723 in the tail.

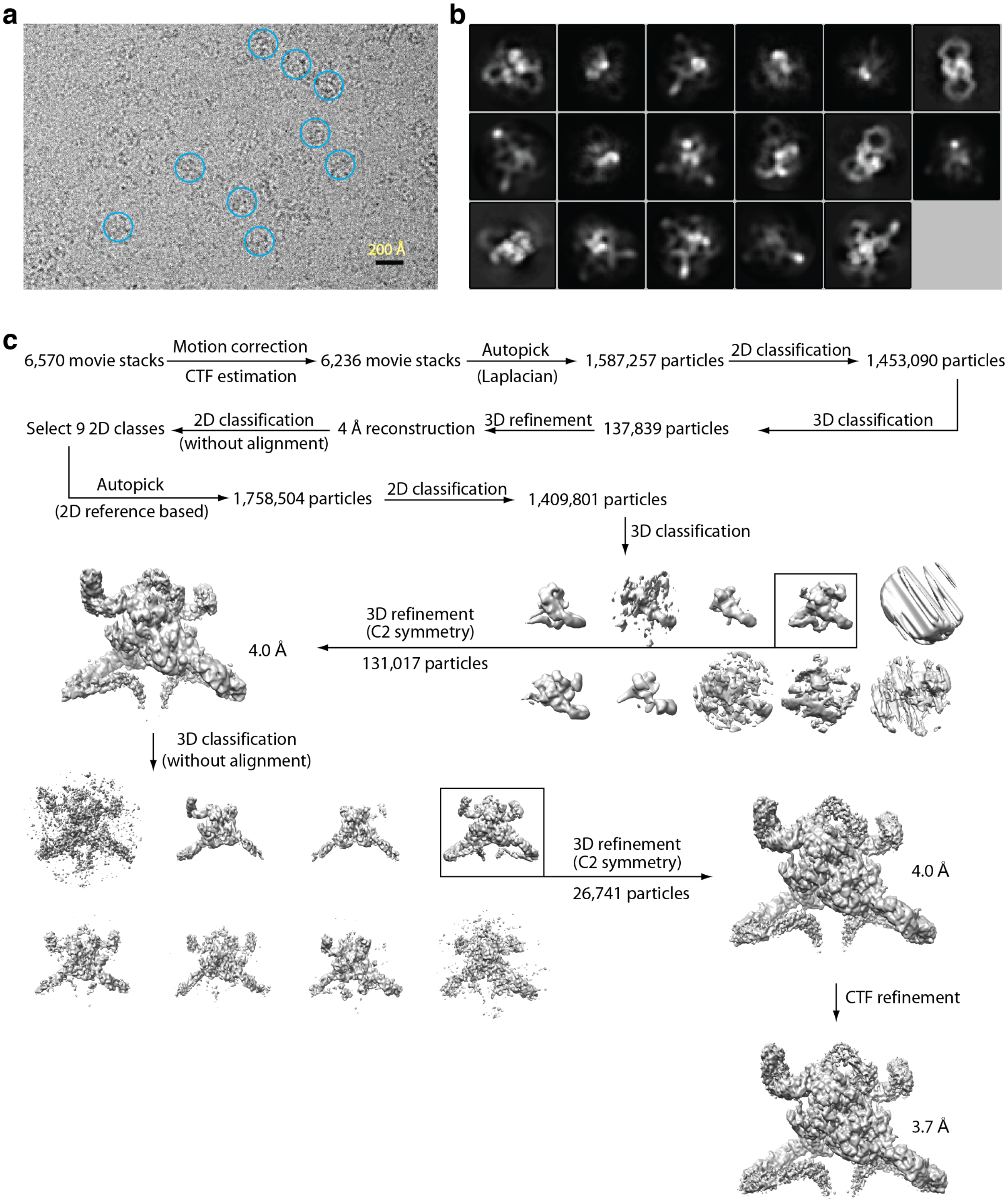


**Supplementary figure 2. Image process procedure**

(a) One representative motion-corrected micrograph from the Cryo-EM dataset. A few particles are highlighted by circles.

(b) 2D class averages of particles used for subsequent 3D reconstruction.

(c) Flowchart of the image processing procedure.

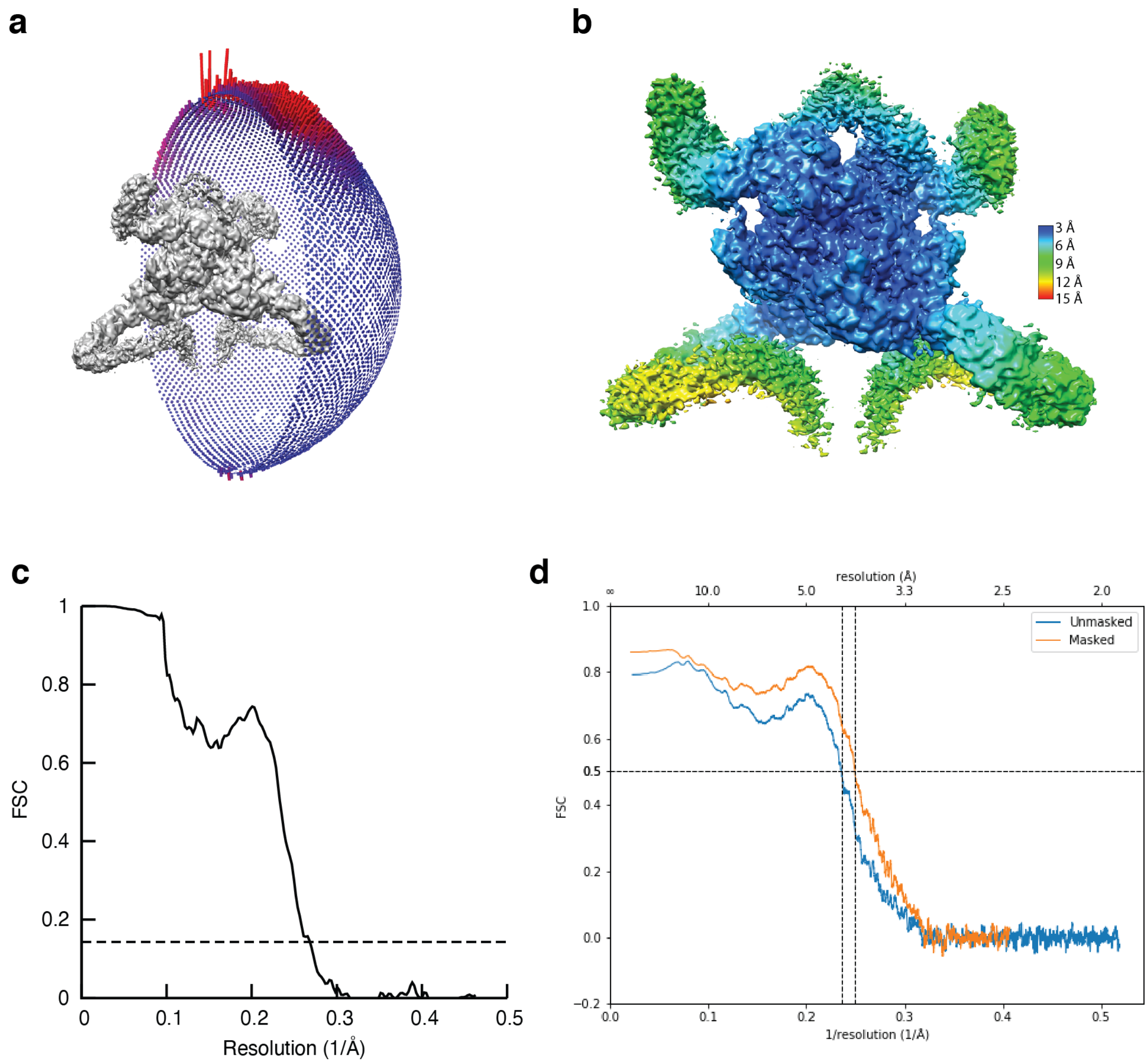


**Supplementary figure 3. Resolution analyses of the cryo-EM structure**
(a) Euler angle distribution of particles used for the 3D reconstruction. Height of rods represents the number of particles.
(b) Local resolutions of the Cryo-EM map.
(c) Gold standard FSC curve of the final reconstructions of the complex. Dashed lines indicate FSC=0.143.
(d) FSC curves between the maps and atomic models of the complex. The horizontal dashed line indicates FSC=0.5.

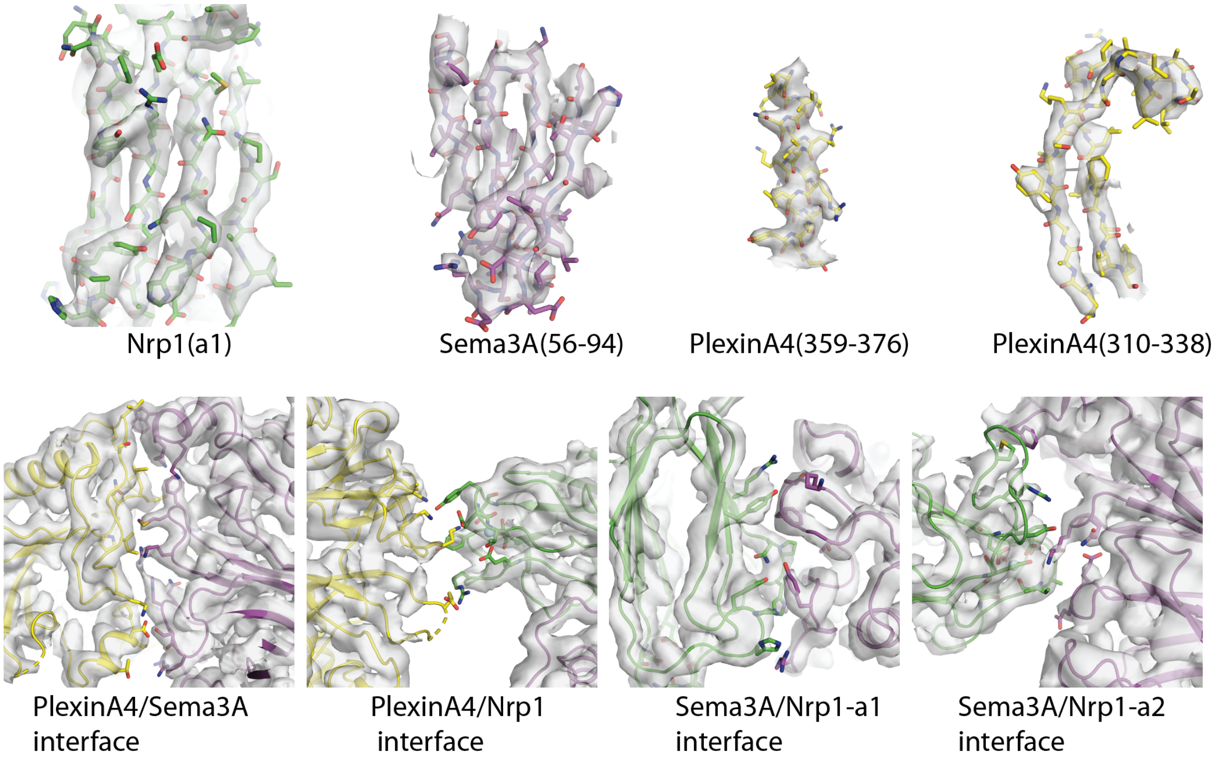


**Supplementary figure 4. Expanded views of density maps of various parts of the structure**

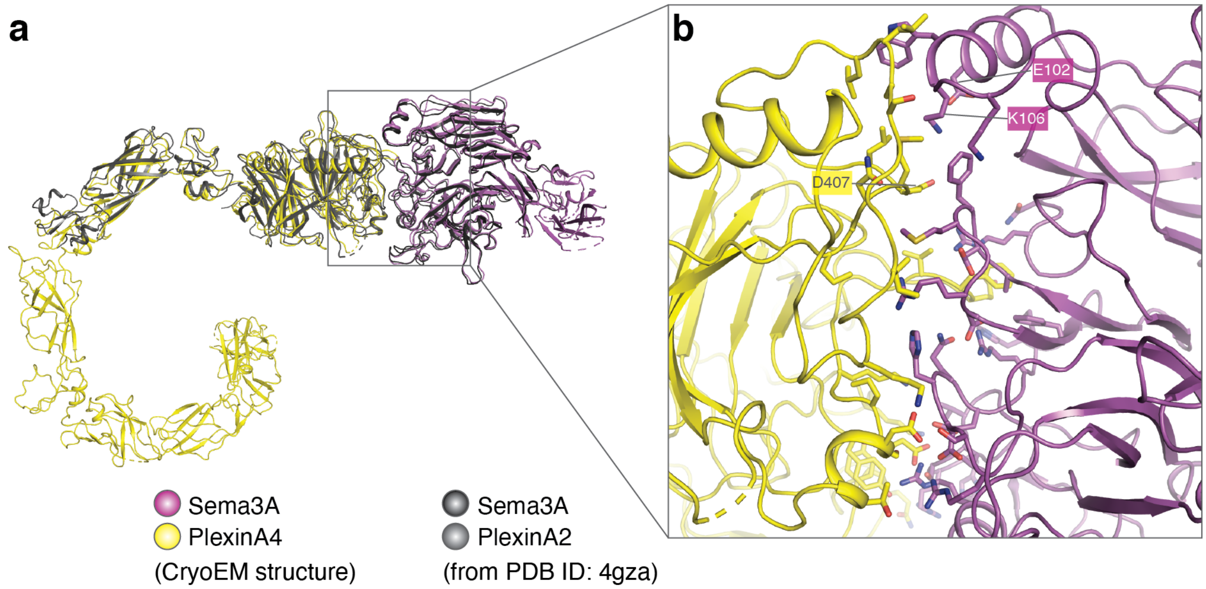


**Supplementary figure 5. Interface between Sema3A and PlexinA4**
(a) Overview of the Sema3A/PlexinA4 binding mode. For clarity, only one Sema3A molecule and one PlexinA4 molecule from the 2:2:2 complex are shown. One PlexinA2/Sema3A complex from PDB ID 4gza is superimposed for comparison.
(b) Expanded view of the interface. The designed A106K mutation in Sema3A is highlighted. Lys106 sits between Glu102 in Sema3A and Asp407 in PlexinA4. These charge complementary interactions enhance the binding affinity between Sema3A and PlexinA4.

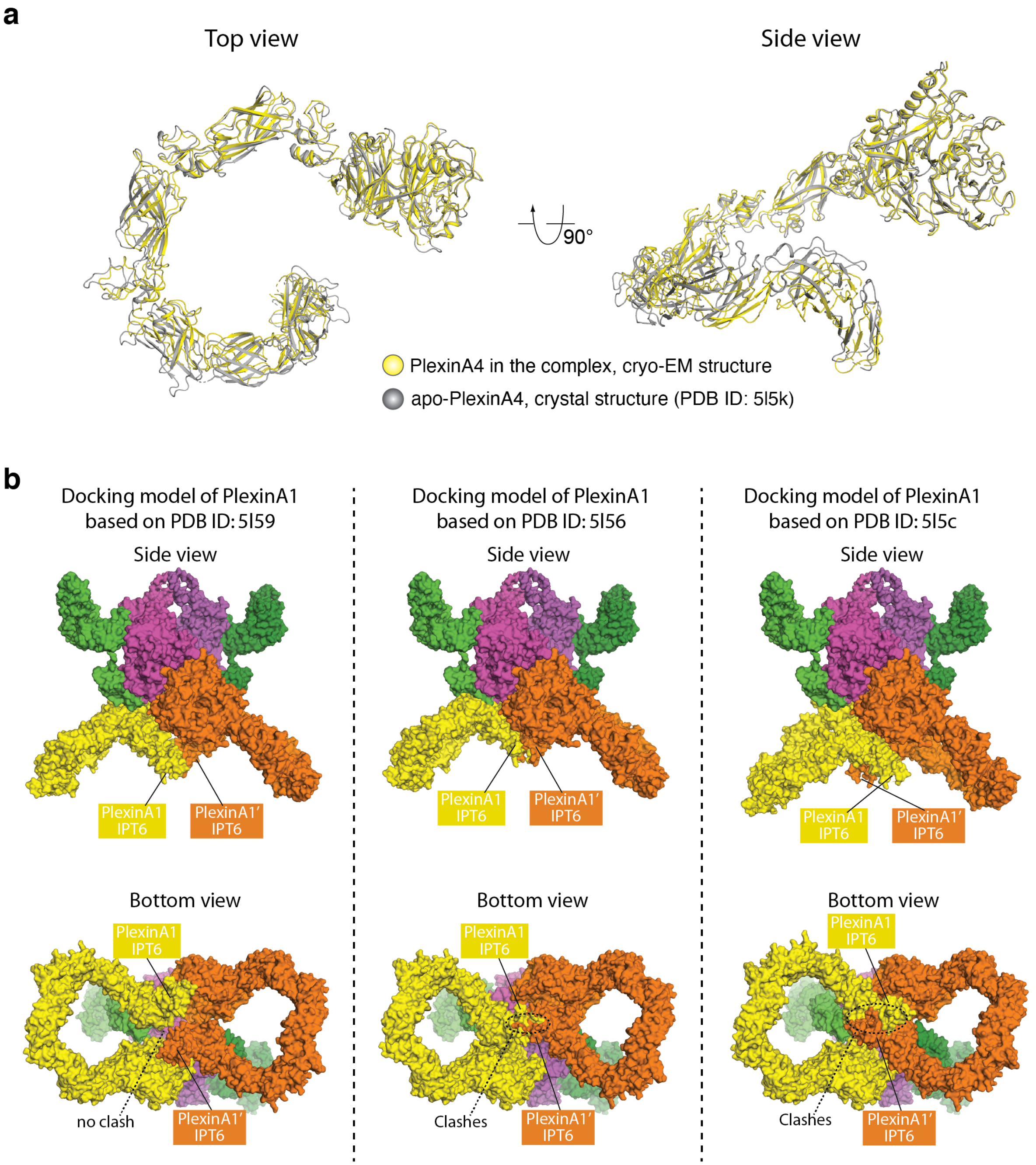


**Supplementary figure 6. Variabilities in the ring-shape of class A plexins**

(a) High degree of similarity between apo-PlexinA4 and PlexinA4 in the 2:2:2 Sema3A/PlexinA4/Nrp1 complex.

(b) Variable conformations seen in three crystal structures of PlexinA1 and its implications in the formation of the 2:2:2 complex. The complex models are generated by docking the crystal structures into the Cryo-EM structure of the 2:2:2 Sema3A/PlexinA4/Nrp1 complex based on the Sema domain of plexin. PlexinA1 from PDB ID 5l59 appears to be able to form the 2:2:2 complex similar to the Sema3A/PlexinA4/Nrp1 complex. In contrast, the models based on the other two PlexinA1 structures show server clashes, especially at the IPT6 domain, indicating that these conformations of PlexinA1 are not compatible with the formation of the 2:2:2 complex.

**a**

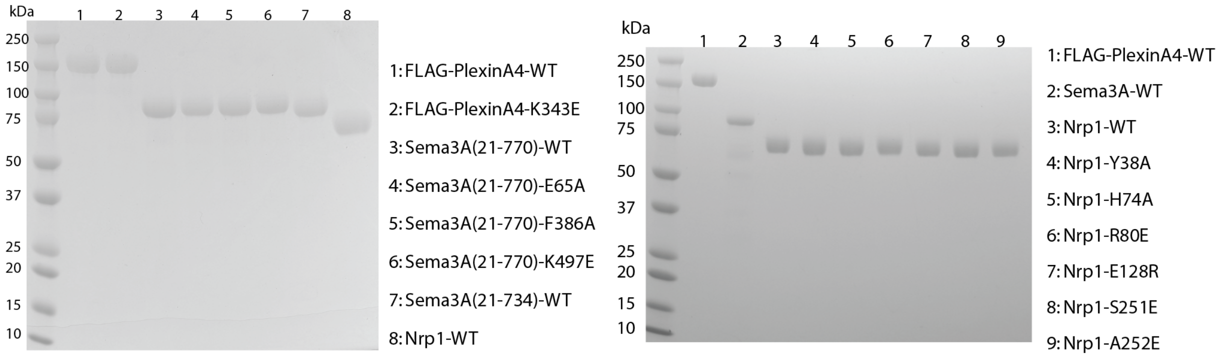


**b**

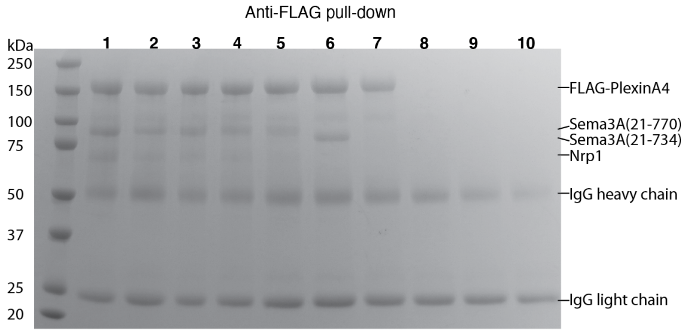


| Lane | Sema3A | FLAG-PlexinA4 | Nrp1 | Interface perturbed | Complex weakened |
|---|---|---|---|---|---|
| 1 | 21-770, WT | WT | WT | - | - |
| 2 | 21-770, WT | K343E | WT | PlexinA4-Sema/Nrp1-a1 | yes |
| 3 | 21-770, E65A | WT | WT | Sema3A-Sema/Nrp1-a2 | yes |
| 4 | 21-770, F386A | WT | WT | Sema3A-Sema/Nrp1-a1 | yes |
| 5 | 21-770, K497E | WT | WT | Sema3A-Sema/Nrp1-a2 | yes |
| 6 | 21-734, WT | WT | WT | Sema3A-tail/Nrp1-b1 | yes |
| 7 | - | WT | - | - | - |
| 8 | 21-770, WT | - | - | - | - |
| 9 | - | - | WT | - | - |
| 10 | - | - | - | - | - |

**c**

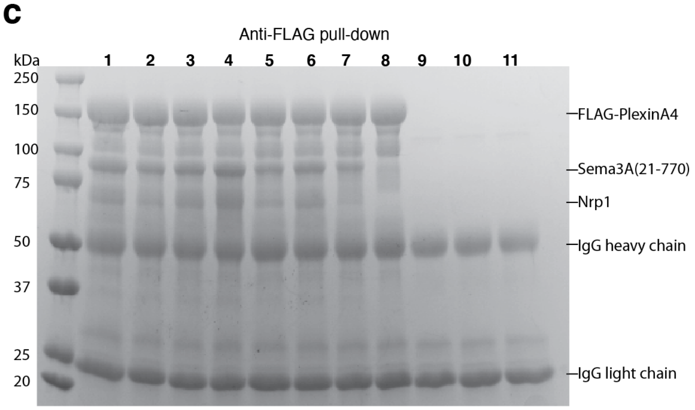


| Lane | Sema3A | FLAG-PlexinA4 | Nrp1 | Interface perturbed | Complex weakened |
|---|---|---|---|---|---|
| 1 | 21-770, WT | WT | WT | - | - |
| 2 | 21-770, WT | WT | Y38A | Sema3A-Sema/Nrp1-a1 | yes |
| 3 | 21-770, WT | WT | H74A | Sema3A-Sema/Nrp1-a1 | no |
| 4 | 21-770, WT | WT | R80E | PlexinA4-Sema/Nrp1-a1 | no |
| 5 | 21-770, WT | WT | E128R | PlexinA4-Sema/Nrp1-a1 | yes |
| 6 | 21-770, WT | WT | S251E | Sema3A-Sema/Nrp1-a2 | no |
| 7 | 21-770, WT | WT | A252E | Sema3A-Sema/Nrp1-a2 | yes |
| 8 | - | WT | - | - | - |
| 9 | 21-770, WT | - | - | - | - |
| 10 | - | - | WT | - | - |
| 11 | - | - | - | - | |

**Supplementary figure 7. Pull-down assay for binding interface mutants of Sema3A, PlexinA4 and Nrp1**

(a) SDS-PAGE analyses of protein samples used in the pull-down assays.

(b) Effects on complex formation of mutations in SemaA3 and PlexinA4. A representative gel from three independent experiments is shown. The results are summarized in the table on the right.

(c) Effects on complex formation of mutations in Nrp1. A representative gel from three independent experiments is shown. The results are summarized in the table on the right.

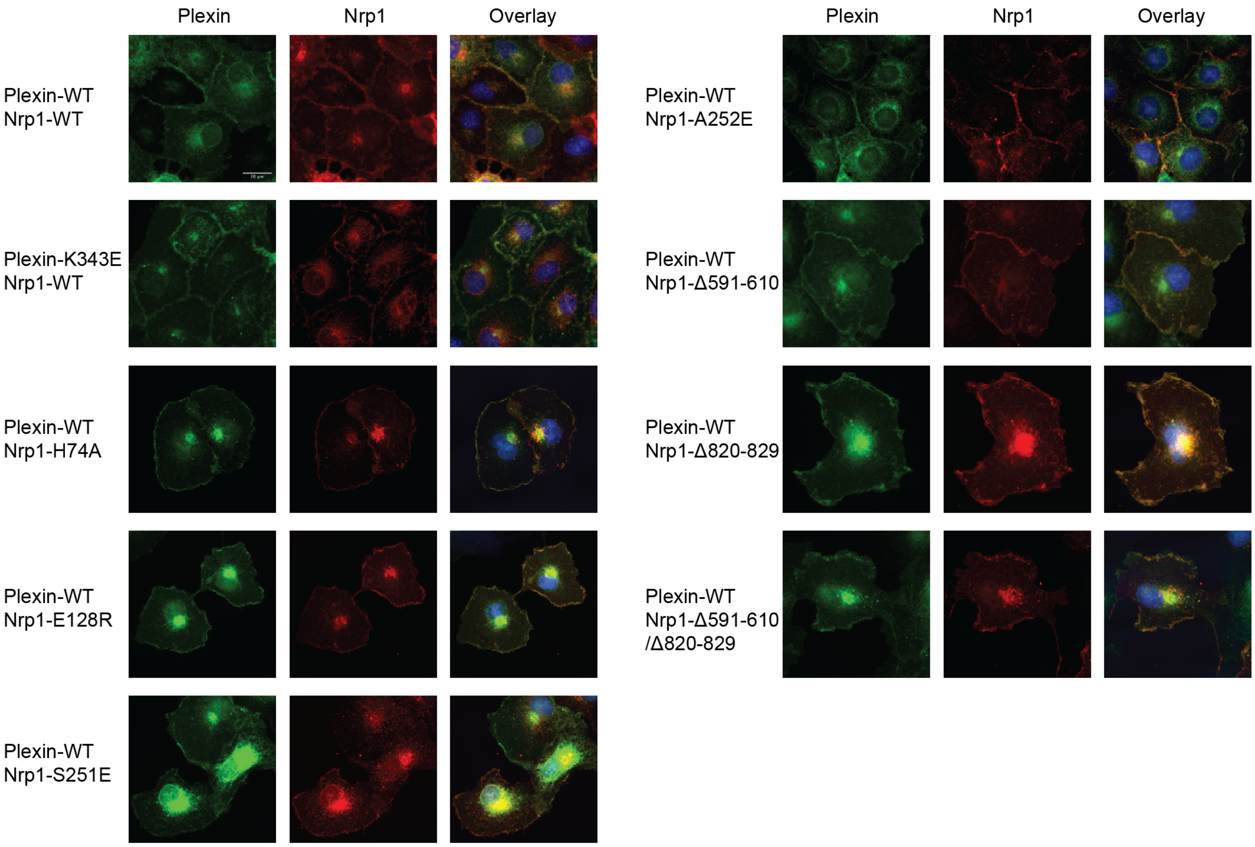


**Supplementary figure 8. Cell surface expression of PlexinA4 and Nrp1.**
COS7 cells stably expressing various combinations of myc-tagged PlexinA4 and FLAG-tagged Nrp1 constructs for the collapse assay were subjected to immunofluorescence imaging. PlexinA4 and Nrp1 were immuno-stained with an anti-myc and anti-FLAG antibody, respectively. Nuclei were stained with DAPI. One representative image from three biological repeats for each cell line is shown. The results show that both the wild type and all the mutants of the two proteins were expressed on the cell surface. Various portions of the proteins remained inside the cell, which present immature proteins undergoing processing in the endoplasmic reticulum and Golgi apparatus. Scale bar represents 20 μm.

**Supplementary Table 1. Primers used in the study**

| primer | sequence | comments |
|---|---|---|
| mPlxA4-pEZTsp-1226-KpnI-gr | GTGGTGATGGGTACCTGGGGCGATGTATACCATCCCAGG | |
| mPlxA4-pEZTsp-33-XhoI-gf | GTAGCTGAACTCGAGAAGCCTTCCTTTGTGACATTCCGA | |
| mPlxA4-K343E-f | TTCTCCAAGGGCCAGGAGCGGAAGATGAAATCT | |
| mPlxA4-K343E-r | AGATTTCATCTTCCGCTCCTGGCCCTTGGAGAA | |
| mPlxA4-pTY-NotI-gf | GCTCTAGAACTAGCCACCGCGGCCGCGCCACCATG CCCTGGAACTGGACTTGCCTGCTC | |
| mPlxA1-pTY-myc-NheI-gr | GAATTGGCCGCCCTAGATGCAGCTAGCTCACAGATCCTCTT CAGAGATGAGTTTCTGCTCGCTGTCTAAGCTCATGAGAGTT AT | |
| mSema3A-pEZTsp-XhoI-21-gf | GTAGCTGAACTCGAG AACTATGCAAACGGAAAGAAC | |
| mSema3A-pEZTsp-KpnI-770R-3T | GTGGTGATGGGTACCTCATCTGGGTGCCCGCTCAAACTC | |
| mSema3A-pEZTsp-KpnI-734R-3T | GTGGTGATGGGTACCTCAGCGTTGCTTTCGGTCCCTTTTC | |
| mSema3A-A106K-f | GATGAATGCAAATGGAAGGGAAAAGATATCCTG | |
| mSema3A-A106K-r | CAGGATATCTTTTCCCTTCCATTTGCATTCATC | |
| mSema3A-E65A-f | ACCTTCCTTCTGGATGCGGAACGGAGTAGACTA | |
| mSema3A-E65A-r | TAGTCTACTCCGTTCCGCATCCAGAAGGAAGGT | |
| mSema3A-F386A-f | TGTCCCAGTAAAACAGCTGGCGGATTTGACTCC | |
| mSema3A-F386A-r | GGAGTCAAATCCGCCAGCTGTTTTACTGGGACA | |
| mSema3A-K497E-f | ATGGAGCTTTCTACTGAACAGCAACAGCTGTAC | |
| mSema3A-K497E-r | GTACAGCTGTTGCTGTTCAGTAGAAAGCTCCAT | |
| mNrp1-pEZTsp22Fnew-XhoI-gf | GTAGCTGAACTCGAGTTCCGCAGCGACAAATGTGGCGGG | |
| mNrp1-pEZTsp588Pnew-KpnI-gr | GTGGTGATGGGTACCAGGTGCTTCCACTTCACAGCCCAG | |
| mNrp1-Y38A-f | ATCGAAAACCCAGGGGCCCTCACATCTCCCGGT | |
| mNrp1-Y38A-r | ACCGGGAGATGTGAGGGCCCCTGGGTTTTCGAT | |
| mNrp1-H74A-f | ATCAACTTCAACCCAGCTTTCGATTTGGAGGAC | |
| mNrp1-H74A-r | GTCCTCCAAATCGAAAGCTGGGTTGAAGTTGAT | |
| mNrp1-R80E-f | TTCGATTTGGAGGACGAAGACTGCAAGTATGAC | |

| | | |
|---|---|---|
| mNrp1-R80E-f | GTCATACTTGCAGTCTTCGTCCTCCAAATCGAA | |
| mNrp1-E128R-f | TTTGTCTCTGACTATAGGACACATGGGGCAGGG | |
| mNrp1-E128R-r | CCCTGCCCCATGTGTCCTATAGTCAGAGACAAA | |
| mNrp1-S251E-f | GTCTTTTACACTGACGAGGCAATAGCAAAAGAA | |
| mNrp1-S251E-r | TTCTTTTGCTATTGCCTCGTCAGTGTAAAAGAC | |
| mNrp1-A252E-f | TTTTACACTGACAGCGAGATAGCAAAAGAAGGT | |
| mNrp1-A252E-r | ACCTTCTTTTGCTATCTCGCTGTCAGTGTAAAA | |
| mNrp1-F-g-NotI | GCTCTAGAACTAGCCACCGCGGCCGCGCCACCATGGAGAG GGGGCTGCCGTTGCTGTGC | |
| mNrp1-R-g-NheI_Flag | GAATTGGCCGCCCTAGATGCAGCTAGCTCACTTATCGTCGT CATCCTTGTAATCCGCCTCTGAGTAATTACTCTGTGGGTTC | |
| mNrp1-del591-610-f | GAAGCACCTACAGCTCACAGTGGCACAGGT | |
| mNrp1-del591-610-r | ACCTGTGCCACTGTGAGCTGTAGGTGCTTC | |
| mNrp1-del820-829-f | CTAGATAAAAAGAACACTCCAGGATATGAA | |
| mNrp1-del820-829-r | TTCATATCCTGGAGTGTTCTTTTTATCTAG | |
