# Supplementary material for: Architecture of the Sema3A/PlexinA4/Neuropilin tripartite complex
Source: Nat Commun. 2021 May 26;12:3172. doi: 10.1038/s41467-021-23541-x (PMC8155012; doi:10.1038/s41467-021-23541-x)
Supplement: Supplementary file 3 — Description of Additional Supplementary Files [file 41467_2021_23541_MOESM3_ESM.pdf]

Description of Additional Supplementary Files

Title: Supplementary Movie 1

Description: Overall assembly of the Sema3A/PlexinA4/Nrp1 complex
