# Supplementary material for: Architecture of the Sema3A/PlexinA4/Neuropilin tripartite complex
Source: Nat Commun. 2021 May 26;12:3172. doi: 10.1038/s41467-021-23541-x (PMC8155012; doi:10.1038/s41467-021-23541-x)
Supplement: Supplementary file 5 — Reporting Summary [file 41467_2021_23541_MOESM5_ESM.pdf]

nature research

Corresponding author(s): Xiaochen Bai, Xuewu Zhang

Last updated by author(s): 04/23/2021

Reporting Summary

Nature Research wishes to improve the reproducibility of the work that we publish. This form provides structure for consistency and transparency in reporting. For further information on Nature Research policies, see Authors & Referees and the Editorial Policy Checklist .

Statistics

For all statistical analyses, confirm that the following items are present in the figure legend, table legend, main text, or Methods section.

n/a	Confirmed	
☐	☒	The exact sample size (*n*) for each experimental group/condition, given as a discrete number and unit of measurement
☐	☒	A statement on whether measurements were taken from distinct samples or whether the same sample was measured repeatedly
☐	☒	The statistical test(s) used AND whether they are one- or two-sided<br>*Only common tests should be described solely by name; describe more complex techniques in the Methods section.*
☐	☒	A description of all covariates tested
☐	☒	A description of any assumptions or corrections, such as tests of normality and adjustment for multiple comparisons
☐	☒	A full description of the statistical parameters including central tendency (e.g. means) or other basic estimates (e.g. regression coefficient) AND variation (e.g. standard deviation) or associated estimates of uncertainty (e.g. confidence intervals)
☐	☒	For null hypothesis testing, the test statistic (e.g. *F*, *t*, *r*) with confidence intervals, effect sizes, degrees of freedom and *P* value noted<br>*Give P values as exact values whenever suitable.*
☒	☐	For Bayesian analysis, information on the choice of priors and Markov chain Monte Carlo settings
☒	☐	For hierarchical and complex designs, identification of the appropriate level for tests and full reporting of outcomes
☒	☐	Estimates of effect sizes (e.g. Cohen's *d*, Pearson's *r*), indicating how they were calculated

Our web collection on statistics for biologists contains articles on many of the points above.

Software and code

Policy information about availability of computer code

Data collection	Images were taken by DeltaVision Core microscope with the built-in softwares softWoRx (7.0) and ZOE fluorescent cell imager as detailed in Methods. Cryo-EM data were collected with the EPU package installed on the Titan Krios microscope.
Data analysis	Statistical analysis was performed using GraphPad Prism 9. Cryo-EM image processing, structure determination and analyses were carried using standard software packages including Relion 3.0 and 3.1, Phenix 1.8 and 1.9, Coot 0.89, Pymol 2.3, Chimera 2.3, Motioncorr2 1.1, GCTF 1.06, Molprobity 4.5. Sequence alignments were rendered with Jalview 2.11.1.3.

For manuscripts utilizing custom algorithms or software that are central to the research but not yet described in published literature, software must be made available to editors/reviewers. We strongly encourage code deposition in a community repository (e.g. GitHub). See the Nature Research guidelines for submitting code & software for further information.

Data

Policy information about availability of data

All manuscripts must include a data availability statement. This statement should provide the following information, where applicable:
- Accession codes, unique identifiers, or web links for publicly available datasets
- A list of figures that have associated raw data
- A description of any restrictions on data availability

The atomic coordinates and cryo-EM map have been deposited into the RCSB (entry ID: 7M0R [https://doi.org/10.2210/pdb7M0R/pdb]) and EMD database (entry ID: EMD-23613 [https://www.emdataresource.org/EMD-23613]) respectively. Source data are provided with this paper. All the relevant data are available from the authors.

Field-specific reporting

Please select the one below that is the best fit for your research. If you are not sure, read the appropriate sections before making your selection.

- [x] Life sciences
- [ ] Behavioural & social sciences
- [ ] Ecological, evolutionary & environmental sciences

For a reference copy of the document with all sections, see nature.com/documents/nr-reporting-summary-flat.pdf

Life sciences study design

All studies must disclose on these points even when the disclosure is negative.

Sample size	For quantification of percentages of cells with phenotype of collapse, random fields were chosen throughout the slide. More than 1000 cells were counted for each sample. The population size was ~1000,000 cells, which was the number of cells seeded in plates. According to an online sample size calculator (https://www.calculator.net/sample-size-calculator.html), 267 cells are needed to have a confidence level of 95% that the real value of collapse percentage within 6% of the counted value. The number of cells counted for any group ranges from 379 to 3020, far exceeding the required number.
Data exclusions	CryoEM data were processed in Relion, which excluded low-quality data to reach high-resolution using statistical methods. The exclusion criteria is pre-established as implemented in Relion, a common practice in cryo-EM
Replication	The cell collapse assay was confirmed with at least three biological replicates as detailed in Methods or Figure Legends. All attempts at replication were successful, and the results are reproducible. No data were excluded. The pull-down experiments were repeated independently three times. The results are reproducible. One representative image for each is shown.
Randomization	Fields of view were randomly chosen throughout the slide of each sample using DAPI channel to avoid bias in selection of cells with particular phenotypes. Each mutation of one of the three proteins (Sema3A, PlexinA4 and Nrp1) was combined with the wild type of the other two proteins in order to test the effect of the mutations on the cell collapse activity. No randomization is needed for this part. The structure determination procedure and other experiments followed standard procedures in the field that do not need randomization.
Blinding	During transfection of cells, the investigators were not blinded to group allocation because we had to know which plasmids were used to make cells that stably expressed the desired constructs. For counting collapsed cells, however, the group allocation were masked and coded with numbers. By doing so, the observers were blinded to identities of the groups of cells to avoid bias during counting collapsed and non-collapsed cells. Structural determination and other experiments in the paper followed standard procedure in the field that do not need blinding.

Reporting for specific materials, systems and methods

We require information from authors about some types of materials, experimental systems and methods used in many studies. Here, indicate whether each material, system or method listed is relevant to your study. If you are not sure if a list item applies to your research, read the appropriate section before selecting a response.

Materials & experimental systems

n/a	Involved in the study
	x Antibodies
	x Eukaryotic cell lines
x	Palaeontology
x	Animals and other organisms
x	Human research participants
x	Clinical data

Methods

n/a	Involved in the study
x	ChIP-seq
x	Flow cytometry
x	MRI-based neuroimaging

Antibodies

Antibodies used	anti-FLAG resin (Sigma, #2220), rabbit anti-myc antibody (Cell signaling, #2278S), mouse anti-myc antibody (Cell signaling, #2276S), Alexa488-conjugated anti-rabbit IgG (Life Technologies, A111034), mouse anti-FLAG (Bimake, A5712), Cy3-conjudated anti-mouse IgG (Invitrogen, A10521), mouse anti-myc antibody (Takara, 631212), Alexa-488-conjugated anti-mouse IgG (Thermo-Fisher, A11029). Dilution factors of the antibodies are described in the paper
Validation	All the commercial antibodies have been verified by the manufacturers. Our negative control experiment validated the specificity of the antibodies used in our study. All the specie specificity, noisy signaling, and application were also validated with positive and negative controls. Three primary antibodies were used in the study:<br>1. Rabbit anti-myc antibody (Cell signaling #2278S); Detailed information can be found at:<br>https://www.cellsignal.com/products/primary-antibodies/myc-tag-71d10-rabbit-mab/2278?site-search-type=Products&N=4294956287&Ntt=2278s&fromPage=plp&_requestid=3031570<br>2. Mouse anti-myc antibody (Cell signaling #2276S); Detailed information can be found at:

https://www.cellsignal.com/products/primary-antibodies/myc-tag-9b11-mouse-mab/2276?site-search-type=Products&N=4294956287&Ntt=9b11&fromPage=plp
3. Mouse anti-FLAG antibody (Bimake, #A5712); Detailed information can be found at:
https://www.bimake.com/antibody/anti-fag-tag-mouse-recombinant-mab.html

Eukaryotic cell lines

Policy information about cell lines

Cell line source(s)	HEK293T and COS7 cells were purchased form ATCC. Expi293F cells were purchased from ThermoFisher Scientific (A14527).
Authentication	The cell lines used in this study were assumed to be authentic by the manufactures based on the manufactures' descriptions, but no details are provided on the manufacture's website. No further authentications were carried out by the authors.
Mycoplasma contamination	The cell lines used in this study were free of mycoplasma contamination based on the results of e-Myco Mycoplasma PCR Detection Kit (Bulldog Bio) and were regularly maintained with Normocin (antimicrobial reagent against mycoplasma, bacteria and fungi) (InvivoGen).
Commonly misidentified lines (See ICLAC register)	No commonly misidentified cells were used in this study.
